# Male sexually coercive behaviour drives increased swimming efficiency in female guppies

**DOI:** 10.1111/1365-2435.12527

**Published:** 2015-08-24

**Authors:** Shaun S. Killen, Darren P. Croft, Karine Salin, Safi K. Darden

**Affiliations:** ^1^ Institute of BiodiversityAnimal Health and Comparative MedicineUniversity of GlasgowGraham Kerr BuildingGlasgowG12 8QQUK; ^2^ Department of PsychologyUniversity of ExeterWashington Singer LaboratoriesExeterEX4 4QGUK

**Keywords:** locomotion, metabolic rate, phenotypic plasticity, sexual conflict, teleost fish

## Abstract

1. Sexual coercion of females by males is widespread across sexually reproducing species. It stems from a conflict of interest over reproduction and exerts selective pressure on both sexes. For females, there is often a significant energetic cost of exposure to male sexually coercive behaviours.
2. Our understanding of the efficiency of female resistance to male sexually coercive behaviour is key to understanding how sexual conflict contributes to population level dynamics and ultimately to the evolution of sexually antagonistic traits.
3. Overlooked within this context are plastic physiological responses of traits within the lifetime of females that could moderate the energetic cost imposed by coercive males. Here, we examined whether conflict over the frequency and timing of mating between male and female guppies *Poecilia reticulata* can induce changes in swimming performance and aerobic capacity in females as they work to escape harassment by males.
4. Females exposed to higher levels of harassment over a 5‐month period used less oxygen to swim at a given speed, but displayed no difference in resting metabolic rate, maximal metabolic rate, maximal sustained swimming speed or aerobic scope compared to females receiving lower levels of harassment.
5. The observed increase in swimming efficiency is at least partially related to differences in swimming mechanics, likely brought on by a training effect of increased activity, as highly harassed females spent less time performing pectoral fin‐assisted swimming.
6. Sexual conflict results in sexually antagonistic traits that impose a variety of costs, but our results show that females can reduce costs through phenotypic plasticity. It is also possible that phenotypic plasticity in swimming physiology or mechanics in response to sexual coercion can potentially give females more control over matings and affect which male traits are under selection.

## Introduction

Over the last century, our interpretation of reproductive interactions between the sexes has changed from one of cooperation to one of probable conflict, that is a disparity in the evolutionary reproductive interests of males and females (Darwin 1871; Parker 1979). Intersexual conflict results in sex differences in the optimal outcome of male–female encounters and the subsequent development of behaviours and adaptations that can benefit one sex while being costly to the other (Parker 1979; Chapman *et al*. 2003). Male sexual coercion of females is one such behavioural outcome and occurs in a range of invertebrate and vertebrate species (Clutton‐Brock & Parker 1995; Chapman *et al*. 2003; Parker 2006). Sexual harassment is a form of coercion where males persistently court, pursue or attempt to mate with unreceptive females. Numerous potential costs of male sexual harassment have been documented, including increased energy expenditure (Clutton‐Brock & Langley 1997), reduced foraging (Magurran & Seghers 1994a), increased predation risk (Rowe 1994), and direct injury or even mortality among females (Chapman *et al*. 2003; Arnqvist & Rowe 2005). Females of some species enact behavioural responses to reduce these costs, for example by selecting habitats with fewer harassing males (Clutton‐Brock, Price & MacColl 1992; Réale, Boussès & Chapuis 1996; Darden & Croft 2008; Croft, Darden & Ruxton 2009) or by forming social structures to fend off male attacks (Agrillo, Dadda & Bisazza 2006; Silk 2007; Brask *et al*. 2012). It is unknown, however, whether females can also display plasticity in physiological traits that reduce the costs of being harassed. Given the strong selective pressures generated by sexual conflict (Arnqvist & Rowe 2005), it is likely that physiology may show adaptive or plastic responses that enhance the ability of females to cope with persistent harassment by males and reduce associated costs.

Female traits associated with energy use and locomotion are especially likely to be prone to plastic or evolutionary change via male harassment. For example, an increased aerobic or anaerobic capacity, or improved maximal locomotory performance, could allow females to resist coercion or avoid males during pursuits. In humans and other animals, prolonged or repeated bouts of physical exercise can improve locomotory performance (Sinclair *et al*. 2014), alter resting metabolic rate (Speakman & Selman 2003), enhance aerobic and anaerobic capacity by increasing mitochondrial densities or the concentrations of aerobic enzymes in muscle tissue (Joyner & Coyle 2008; Booth, Roberts & Laye 2011), and even increase the efficiency of movement (Joyner & Coyle 2008). Sexual coercion or harassment in animals often involves frequent and prolonged bouts of intense physical activity associated with altercations or chases or generally higher levels of movement (Lauer, Sih & Krupa 1996; Linklater *et al*. 1999; Sundaresan, Fischhoff & Rubenstein 2007; Jacoby, Busawon & Sims 2010). As such, harassment could constitute an ecological context in which a training effect of repeated physical activity may manifest, generating intraspecific variation in traits associated with locomotory ability and energy use. This is especially likely given the advantages that could be gained by individuals that are able to reduce the costs of harassment (Magurran & Seghers 1994b). Individuals of some species can also show variation in their manner of movement (e.g. changes in gait) that improve locomotory efficiency (Hoyt & Taylor 1981; Svendsen *et al*. 2013). We propose that the physical exercise involved with sexual harassment in nature may act to alter metabolic traits, movement mechanics and, ultimately, levels of physical performance in females that could reduce the costs of harassment.

We examined the potential plasticity of physiological traits in female Trinidadian guppies (*Poecilia reticulata*) reared in varying sociosexual environments. The Trinidadian guppy is a sexually dimorphic freshwater fish that within its native range inhabits riffle and pool habitats (Magurran 2005). In the wild, male guppies spend a large part of their time budget attempting to mate with females, with females in some populations receiving more than one coercive mating attempt per minute (Magurran & Seghers 1994b). Harassment by male guppies can consist of repeated bouts of courtship, chasing and coerced mating attempts, all of which the females can attempt to avoid by employing burst‐type swimming or prolonged bouts of high‐speed swimming during pursuits. Work in this species has documented an energetic cost in the form of reduced energy intake as a result of male sexually coercive behaviour (Magurran & Seghers 1994a), but nothing is known about whether females can adjust their physiology to minimize locomotory costs stemming from increased harassment. Here, we test the hypothesis that prolonged exposure to male harassment in female guppies can generate increased swimming performance. Furthermore, we examine the mechanisms underlying any changes in physical performance by quantifying swimming costs, aerobic and anaerobic capacity, resting metabolic rate, muscular aerobic enzyme activity (citrate synthase), swimming mechanics and morphology in females that had been housed with higher or lower levels of harassment by males over a period of several months.

## Materials and methods

### Animal Holding and Exposure to Harassment

We used laboratory‐reared Trinidadian guppies descended from wild fish collected from the lower reaches of the Aripo River (10°40′N 61° 14′W) on the island of Trinidad in 2008. Fry were collected from laboratory stock pools (150 × 300 cm) and reared in gravel‐bottomed tanks (30 × 30 × 45 cm) on a flow‐through system in groups of 30 until they began to show sexual differentiation (*c*. 8 weeks). At this point, juveniles were sorted into groups of 20 in one of two male: female sex ratios: (i) high male (1 : 1, HM) and (ii) low male (1 : 5·7, LM). These sex ratios are consistent with those found in the wild (Magurran 2005) and were used to manipulate the levels of sexual harassment experienced by females. Fish were housed in these rearing groups in 30 × 30 × 45 cm tanks (12 tanks per treatment) on a flow‐through system for 5 months (three parturition events). The flow‐through system ensured that fish experienced the same water chemistry during the rearing period. This work was done as approved by the UK Home Office under Project Licence No. 60/4461.

While fish were in month 5 of their rearing treatment conditions, we measured female activity levels in their home tanks (*n* = 10 HM groups, *n* = 11 LM groups). Tanks were divided into eight equal‐sized three‐dimensional zones (15 × 10·5 × 22·5) using white gravel lines and pen marks on the front of the aquaria (four zones in the upper water column and four zones in the lower water column). Two focal females were selected from each tank (*n* = 20 HM females, *n* = 22 LM females) and observed sequentially for 4 min by a single, naïve observer, to record their zone use and swimming behaviour (occurrence of swim bursts) using a hand‐held PDA (Palm Tungsten) with FIT‐System software (Held & Manser 2005). Observations were carried out prior to the first feed of the day by a single observer who was blind to the hypothesis being tested as well as the overall aim of the experiment. We tested for an effect of treatment on the number of zone changes (number of times focal fish entered a new zone in the tank) and swim bursts a focal female made in the 4‐min observation period.

Prior to taking physiological measures, females were removed from their rearing groups and sorted into groups of six unfamiliar, same treatment females (18 groups of each sex ratio origin) and again housed in the flow‐through system. They were left in these all‐female groups for a period of 18 days prior to testing. Given that harassing males have been shown to decrease the foraging efficiency of females, this period of time allowed us to standardize not only the immediate social environment and types of social stressors experienced by focal females, but also the overall body condition of females tested. Throughout the rearing and housing periods, fish were fed *ad libitum* twice daily on a diet of newly hatched shrimp (*Artemia* sp.) and commercial tropical fish flake.

### Swimming Performance and Oxygen Consumption

The swimming performance and oxygen uptake of fish during exercise were quantified using a 170‐mL Blazka‐type swim tunnel (10 cm length × 2·64 cm diameter; volume Loligo Systems, Tjele, Denmark). Fish were held without food for 24 h prior to the beginning of experimental procedures. An individual fish would then be netted from their holding tank, measured for standard length (HM: 2·61 ± 0·05 cm; LM: 2·52 ± 0·05 cm) and mass (HM: 0·330 ± 0·025 g; LM: 0·335 ± 0·041 g). The treatment to be measured in a given trial (HM or LM) was randomly determined by coin flip (*n* = 10 fish tested per treatment). To initiate a trial, fish were carefully place into the swim tunnel without air exposure. Water speed [body lengths (BL) s^−1^] within the tunnel was manipulated with an external control box and voltmeter, which could be used to control the motor and impellor. Prior to experiments, water speeds were calibrated by filming the flow of injected red dye within the tunnel at seven different voltage settings, corresponding to a water speed range of 9–38 cm s^−1^. After being placed into the swim tunnel, fish were exposed to a brief ‘orientation’ trial in which the speed was gradually increased to three BL s^−1^ and then lowered to 2 BL s^−1^ (the slowest speed at which fish consistently orient towards the current). This was repeated 2–3 times, allowing the fish to become familiar with the tunnel and accustomed to facing into the flow of water. The water speed was then set to 2 BL s^−1^. The tunnel was set within an aerated water bath, with the temperature maintained at 25·0 ± 0·1 °C with a thermostat that controlled a flow of water through a stainless steel coil within a heated reservoir as required. To minimize disturbance, the bath was covered in black polystyrene, with the exception of an opening at the top which allowed a small amount of ambient light to enter the test area. This allowed the fish to visually orient itself within the tunnel and permitted the experimenter to see the fish while standing away from the set‐up the using a mirror mounted at a 45° angle adjacent to the tunnel. To help reduce stress for the guppies, the tunnel was surrounded by plastic plants placed within the water bath. In addition, a strip of black polystyrene was fixed around the upstream portion of the tunnel which further encouraged the fish to swim against the current as they displayed a preference towards swimming within this shaded region of the tunnel.

While fish were in the swim tunnel, water oxygen content was quantified once every 2 s using a Firesting 4‐channel oxygen meter and associated sensors (PyroScience GmbH, Aachen, Germany), with the optode inserted horizontally through the upstream end of the swim tunnel. Using a digital recycling timer (SuperPro Technology Ltd., Xiamen, China), a flush pump connected to the swim tunnel was set for alternating periods of 2 min on and then 6 min off. When the pump was switched on, aerated water would be pumped into the swim tunnel and out a small vent open to the air. When the pump was off, the swim tunnel was effectively sealed, and the measures of oxygen content within the tank could be used to calculate the oxygen consumption of the fish swimming within the tunnel. After placement into the tunnel and the brief orientation session, fish were left undisturbed for the next 6 h while swimming at 2 BL s^−1^. Pilot experiments, in which fish were left overnight for 15 h, demonstrated that after placement in the tunnel, fish reached a stable level of oxygen uptake within 2 h. After the 6‐h acclimation period, water speeds were gradually increased at 1·0 BL s^−1^ increments. At each speed, two measurements of oxygen uptake were recorded (i.e. two 2‐min flush pump ‘on’ and two 6‐min ‘off’ phases, meaning each step lasted 16 min). Water speeds within the tunnel were corrected for solid blocking effects (Bell & Terhune 1970). The test continued in this manner until the fish could no longer maintain station within the tunnel and came into contact with the downstream retaining grid for consecutive 10 s. At this point, the water speed was returned to 2 BL s^−1^, and the flush pump was turned off for 20 min, in order to record oxygen uptake during the immediate recovery phase for the calculation of excess post‐exercise oxygen consumption (EPOC). The gait transition speed (*U*_gt_) was defined as the speed increment at which fish first displayed a transition from aerobic steady‐state swimming to the inclusion of burst‐type, anaerobically powered unsteady swimming. The critical swim speed (*U*_crit_) of each fish was calculated as described by Brett (1965) (Brett 1965): $U_{\mathrm{crit}}=U_{f}+t_{f}{\left(t_{s}U_{s}\right)}^{-1}$, where *U*_*f*_ is the water velocity of the penultimate stepwise increase, *t*_*f*_ is the time spent swimming at the final step, *t*_*s*_ is the time of each full step, and *U*_*s*_ is change in water velocity with each step. The maximal metabolic rate (MMR) of each fish was taken as the maximum rate of oxygen uptake recorded throughout the test. Standard metabolic rate (SMR) was estimated as the y‐intercept of a semi‐log plot of oxygen uptake versus speed (only including speeds below which anaerobic metabolism was playing a major role, as indicated by a levelling off of oxygen uptake with further increases in speed). Aerobic scope (AS) was calculated as the difference between MMR and SMR. EPOC for each individual was estimated by calculating the area under the sixth‐order polynomial recovery function (above the baseline cost of swimming at 2 BL s^−1^), until the time at which fitted values were equal to mean level of oxygen uptake while swimming at 2 BL s^−1^ in the 2 h immediately before the beginning of the swim test. EPOC represents the increase in oxygen consumption above routine levels occurring during recovery from a bout of exhaustive anaerobic exercise and represents the anaerobic capacity of an animal (Gastin 1994; Lee *et al*. 2003; Killen *et al*. 2014).

The gross cost of transport (COT) per unit distance (in J kg^−1^ cm^−1^) was calculated for each fish by first converting oxygen uptake at each swim speed to calories using an oxycaloric coefficient of 3·24 cal g $O_{2}^{-1}$ (Claireaux, Couturier & Groison 2006) and then converting calories to Joules (4·18 J cal^−1^). This value was then divided by the corresponding speed (in cm s^−1^). A second‐order quadratic regression was then fitted to the relationship between swim speed (in BL s^−1^) and gross cost of transport. The speed at which the minimum fitted value for gross COT is achieved was considered to be the optimum swim speed for an individual (*U*_opt_). Net COT – the energy expenditure solely due to physical activity above maintenance functions – was also calculated for each individual by initially subtracting SMR from the oxygen uptake at each swim speed and then calculating COT as previously described.

### Swimming Mechanics

To further investigate the mechanisms underlying differences in swimming efficiency between LM and HM females, additional swimming trials were conducted to examine differences in swimming mechanics among female fish from each treatment receiving different levels of male harassment. These experiments were done on a separate set of individuals (*n* = 8 per treatment) from a separate cohort of females that had been subjected to the same harassment treatments as previously described. Individuals were subjected to a *U*_crit_ test as previously described, except there was only one 6‐min interval at each speed increment with a 2‐min ‘acclimation’ period at the beginning of each stepwise increase. All trials were video‐recorded from above high‐definition video camera (Sony Handycam HDR XR260; Sony Corporation, Tokyo, Japan). Videos were later analysed blind to determine the amount of time each individual spent using pectoral fin movements to aid in swimming, as described by Svendsen *et al*. (2013).

### Enzyme Analysis

Females from the same cohort reared for the study of swimming mechanics were also measured for muscle citrate synthase (CS) activity. Females (HM: *n* = 20; LM: *n* = 15) were killed with an overdose of anaesthetic, and the skeletal muscle was removed with the aid of a binocular microscope with the fish on a cooled surface. The samples were immediately frozen in liquid nitrogen and then stored at −80 °C until analysis. The CS assay was adapted from Frazier & Thorburn (2012). Briefly, the frozen muscle was homogenized with a grinding mill for 40 s, 20 Hz, at a concentration of 200 mg wet tissue per mL buffer (5 mm HEPES, 1 mm EGTA, 210 mm d‐mannitol, 70 mm sucrose and pH 7·2 at 4 °C). A pilot study was done to optimize protocol of durations and intensities of homogenization (on brown trout – data not shown here). The homogenate was centrifuged at 800 *g*, 10 min, 4 °C, and then, the supernatants were flash‐frozen and stored at −70 °C until the enzymatic assay. Enzymatic activity was assessed for 15 min spectrophotometrically at 25 °C, in duplicate, on microplate and with shaking. Muscle homogenate was assessed at a concentration of 5 mg mL^−1^, in a KPi buffer (50 mm, pH 7·4) pre‐equilibrated at 25 °C. Protocol of preparation of tissue homogenate in mammals included a cycle of freezing, thawing and then sonification of the homogenate supernatants. Pilot experiments demonstrated that these steps did not improve enzymatic activity in fish tissues and in fact resulted a trend towards reduced enzyme activity. The CS activity was monitored as the rate of generation of thionitrobenzoate anion by measuring the absorbance at 412 nm. The reaction was initiated by adding 0·1 mm acetyl CoA and 0·1 mm oxaloacetic acid. For each sample, blank activity was evaluated by running in parallel the assay without oxaloacetic acid. Determination of the protein content of muscle was performed using a bicinchoninic acid assay, where bovine serum albumin was used as standard and absorbance was read at 562 nm. The results are calculated as the rate of production of thionitrobenzoic acid (μm min^−1^ mg^−1^ of protein) using an extinction coefficient of 13·6.

### Statistical Analysis

All analyses were performed with SPSS statistics v20.0 (SPSS Inc. and IBM, Chicago, IL, USA). The level of significance for all tests was α = 0·05. For all statistical models, plots of residual versus fitted values were inspected to ensure that the assumptions of normally distributed and homogenous residuals were fulfilled. Differences in the routine swimming behaviour of LM and HM females while in stock tanks being exposed to male harassment were analysed using general linear models (GLMs) with either the number of bursts per minute or zone changes as the dependent variable and harassment level as a fixed factor. Difference in metabolic traits were analysed using GLMs with either SMR, MMR, AS, FAS, EPOC, citrate synthase activity or red muscle protein content as the dependent variable, harassment level as a fixed effect and body mass as a covariate (to control for the effects of mass on metabolic traits). Differences in swimming performance indices were analysed using GLMs with either *U*_crit_, *U*_opt_ or *U*_gt_ as a dependent variable, harassment level as a fixed factor and length as a covariate (to control for the effects of body length on swim speed). Differences in log MO_2_, gross COT, log net COT and pectoral fin use were analysed using linear mixed effect models (LMEs) with harassment level as a fixed effect, body mass and speed as covariates and fish ID as a random effect. For gross COT, the quadratic term speed^2^ was also included in the model to account for the curvilinear nature of changes in gross COT with changes in swim speed (Sokal and Rohlf 1995). In all cases, interactions between treatment and speed (and speed^2^) were initially included in LMEs but were removed when non‐significant and the models rerun. To examine potential differences in morphology between treatments that could explain differences in swimming costs, GLMs were employed with either body depth (at the abdomen and also trunk behind the dorsal fin), caudal length and height, caudal surface area or caudal aspect ratio as a fixed effect, harassment level as a fixed effect and total body length as a covariate.

## Results

### Behaviour

While in holding tanks, HM females were 1·9‐fold more active than LM females (Table 1; GLM, effect of harassment, *F*_1,42_ = 5·159, *P* = 0·029). They also performed more than twice as many burst swimming movements per minute as a result of being chased by males (Table 1; 1·06 ± 0·42 for HM; 0·48 ± 0·40 for LM; GLM, effect of harassment, *F*_1,42_ = 21·348, *P* = 0·001).

**Table 1:** Comparisons of routine behaviours, metabolic traits and indices of swimming performance in female guppies reared for several months with exposure to varying levels of male harassment (*n* = 10 per treatment)

Variable	Low harassment	High harassment	F	*P*
Behaviour				
Bursts (min^−1^)	0·477 ± 0·407	1·063 ± 0·420	21·35	<0·001
Zone changes	7·046 ± 6·883	13·700 ± 11·703	5·159	0·029
Metabolic traits				
SMR (mg h^−1^)	0·046 ± 0·025	0·033 ± 0·021	1·539	0·232
MMR (mg h^−1^)	0·312 ± 0·135	0·282 ± 0·093	2·915	0·105
AS (mg h^−1^)	0·258 ± 0·134	0·249 ± 0·085	0·210	0·653
FAS	9·322 ± 7·158	10·727 ± 5·060	0·311	0·585
EPOC (mg O_2_)	0·021 ± 0·013	0·012 ± 0·009	2·839	0·111
Citrate synthase (μm min^−1^ mg^−1^ protein)	142·447 ± 23·523	149·636 ± 31·53	10·549	0·464
Swimming indices				
*U*_crit_ (BL s^−1^)	8·283 ± 1·820	8·963 ± 1·315	1·177	0·293
*U*_gt_ (BL s^−1^)	5·400 ± 0·966	7·100 ± 0·875	15·025	0·001
*U*_opt_ (BL s^−1^)	5·044 ± 1·061	4·892 ± 1·580	0·007	0·936
Minimum COT (J cm^−1^)	0·039 ± 0·005	0·031 ± 0·016	1·450	0·245

SMR, standard metabolic rate; MMR, maximal metabolic rate; AS, aerobic scope; FAS, factorial aerobic scope; EPOC, excess post‐exercise oxygen consumption; U_*crit*_, critical swim speed; U_*gt*_, gait transition speed; U_*opt*_, optimal swim speed; COT, cost of transport; BL, body lengths.

### Swimming Performance and Metabolic Traits

**Figure 1:**
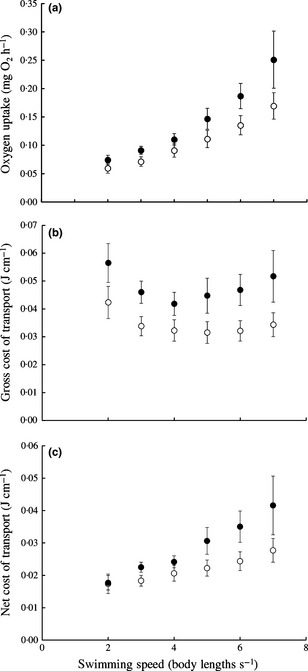
Comparisons of (a) oxygen uptake per unit time; (b) gross cost of transport per unit distance; and (c) net cost of transport per unit distance, over a range of swimming speeds for female guppies reared for 5 months with exposure to varying levels of male harassment (*n* = 10 per treatment). Filled circles = low harassment females; open circles = high harassment females. Data are shown up to the speed at which all fish were engaging in steady‐state aerobically powered swimming (below *U*_gt_ for all fish). Error bars = SEM.

HM females consumed less oxygen while swimming at a given speed (Fig. 1a; LME, effect of harassment, *F*_1,170·84_ = 5·359, *P* = 0·022), and although total AS did not differ between HM and LM females (Table 1), HM females had a greater proportion of total AS available across speeds after accounting for swimming costs (Fig. S1; LME, effect of harassment, *F*_1,791·75_ = 4·385, *P* = 0·037). HM females also spent less energy to move per unit distance (Fig. 1b and c; LME for gross COT, effect of harassment, *F*_1,77·14_ = 6·926, *P* = 0·01; LME for net COT, effect of harassment, *F*_1,60·11_ = 5·297, *P* = 0·025). Minimum COT tended to be higher in LM females, but this difference was not significant (GLM, effect of harassment, *F*_1,17_ = 1·450, *P* = 0·245).

HM and LM females did not differ with respect to SMR, MMR, AS, FAS or EPOC (Table 1; GLM, effect of harassment, *P* > 0·05 in all cases). Further, *U*_crit_ and *U*_opt_ did not differ between treatments (Table 1; GLM, effect of harassment, *P* > 0·05). However, HM females had a higher *U*_gt_ than LM females (Table 1; GLM, effect of harassment, *F*_1,17_ = 15·025, *P* = 0·001).

### Aerobic Enzyme Activity

Skeletal muscle CS activity did not differ between HM and LM females (Table 1; GLM, effect of harassment, *P* > 0·05).

### Swimming Mechanics

**Figure 2:**
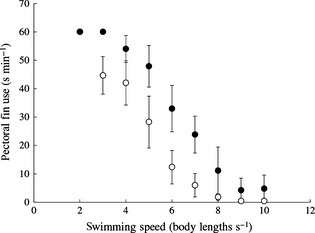
Comparison of the amount of pectoral fin use while swimming at various speeds for females guppies reared for 5 months with exposure to varying levels of male harassment (*n* = 8 per treatment). Filled circles = low harassment females; open circles = high harassment females. Error bars = SEM.

HM females spent less time swimming with pectoral fins extended across speeds (Fig. 2; LME, effect of harassment, *F*_1,10·04_ = 5·726, *P* = 0·038). Across both LM and HM females, fish that used pectoral fins more often had a lower *U*_crit_ (Fig. S2; GLM, effect of pectoral fin use, *F*_1,12_ = 63·16, *P* = 0·001). There were no differences in morphology (e.g. body shape, fin size) between LM and HM females (Table S1).

## Discussion

Our results demonstrate that changes in the locomotory physiology and mechanics of individuals can result from prolonged exposure to sexually coercive tactics. Specifically, female guppies that had experienced higher levels of harassment from males, and thus exhibited higher levels of locomotor activity, swam more efficiently, spending less energy to move at a given speed and had a lower cost of transport than females experiencing lower levels of harassment. Although sexual conflict comes with a variety of costs (e.g. increased energy expenditure, reduced foraging), the results here suggest that females can modulate energetic costs in a way that has not been documented previously.

This study provides the first example in any non‐human animal species of a persistent increase in the efficiency of movement in response to social or ecological pressures. While it has previously been shown that animals moving in groups can position themselves relative to group mates to reduce the costs of locomotion (Killen *et al*. 2012; Marras *et al*. 2015), here it seems that the prolonged increase in high‐intensity swimming caused by harassment can cause plastic changes in the physiology or swimming mechanics of individual fish that reduce costs of movement. The observed pattern of increased efficiency of movement for a given speed shows remarkable similarities to the improved exercise economy observed among human athletes with training (Jones & Carter 2000; Joyner & Coyle 2008). The mechanisms that permit increased efficiency during exercise are not entirely clear, but in human athletes, increased economy of movement can be affected by the type of muscle fibres being recruited and their shortening velocity (Pette 2001; Joyner & Coyle 2008), and possibly the mechanics of the movements themselves (Jones & Carter 2000). Changes in gait during movements are also known to affect the costs of locomotion in a variety of taxa (Hoyt & Taylor 1981). LM females spent more time using pectoral fin movements while swimming, and across both treatments, fish that displayed more pectoral fin use during aerobic swimming had a lower *U*_crit_. Previous work in guppies has demonstrated that intraspecific variation in use of the pectoral fins during swimming is associated with increased energy expenditure at a given speed (Svendsen *et al*. 2013). Our results suggest that the presence of male harassment within a guppy population can alter the degree of variation in swimming mechanics present among females, thus decreasing their costs of locomotion. The actual energetic cost of pectoral fin movements during subcarangiform swimming is relatively small (Gerry & Ellerby 2014), but the pectoral fins are used to stabilize the fish during forward movement, and excessive fin movement is probably a general indicator of suboptimal and apparently inefficient swimming mechanics (Svendsen *et al*. 2013). We found no evidence for plasticity in the capacity for aerobic metabolism at either the whole‐animal (MMR, AS) or biochemical level (i.e. CS activity) in response to varying levels of harassment. However, it is plausible that differences in mitochondrial efficiency could also contribute to the observed differences in locomotory costs (van den Berg *et al*. 2010). Additional work is required to explore this possibility.

Another advantage of increased locomotory efficiency is that an animal will use a lower proportion of their total AS during movement at a given speed. Aerobic scope is the total capacity for oxygen‐consuming physiological tasks above those required for maintenance, including somatic or gonadal growth, digestion and homoeostatic responses to environmental stressors (Claireaux & Lefrancois 2007; Killen *et al*. 2007; Clark, Sandblom & Jutfelt 2013). So, although HM and LM females had a similar total aerobic scope, after the costs of routine swimming are taken into account, HM females had a greater AS available for other physiological functions beyond swimming. For example, when swimming at 7 BL s^−1^, LM females only possessed about 28% of their total AS for tasks other than activity, while HM females had *c*. 43% of their AS remaining. HM females also had a higher *U*_gt_, indicating that they are able to swim at a faster speed before employing burst‐type anaerobic swimming to assist in forward movement. It is therefore conceivable that HM females could possess an increased capacity for coping with fluctuations in environmental parameters such as oxygen availability or temperature while performing routine swimming activity (e.g. due to interactions with conspecifics or in response to stream flow), factors which can constrain AS in ectotherms (Fry 1971; Claireaux & Lefrancois 2007).

Aside from the direct energetic benefits, plasticity in swimming physiology or mechanics could allow females to have more control over reproduction by enhancing their ability to avoid males and resist coerced matings, plus reduce the costs associated with these behaviours. It could be that the increased efficiency of female resistance to mating has a significant effect on the ability of females to persist under the pressures exerted by sexually harassing males. This is a key point as demonstrated by recent theoretical work showing that this efficiency of resistance is a central component in determining population level responses to costly male sexually coercive behaviour, including probability of extinction (Rankin, Dieckmann & Kokko 2011). Increased efficiency of swimming may contribute to costs of harassment behaviour in males if females become more effective in evading males. Therefore, the role of female choice in determining which male traits are under selection could vary within and across populations in response to the level of male harassment present (Magurran & Seghers 1994b). There is the possibility that males also exhibit plasticity in swimming efficiency and improve their ability to pursue females that are better swimmers. In this case, we would expect there to be sexually antagonistic selection on plasticity in swimming performance or efficiency. Plasticity could also lead to differing selection on female phenotypes. For example, changes in swimming ability could alter vulnerability to predation or foraging efficiency.

Female guppies in the wild can employ a number of behavioural tactics to reduce attention from males, such as moving to habitats that males are less likely to occupy (Darden & Croft 2008) or shoaling with more sexually attractive females (Brask *et al*. 2012). Access to larger and more complex habitats may also generally enhance the opportunity for females to avoid male coercive behaviours. Despite this, however, female guppies in the wild can experience more than one unsolicited mating attempt per minute (Magurran & Seghers 1994b), which suggests that the effect we report here is likely to be an important mechanism in the field. Although the current study was performed in a laboratory setting, the experimental conditions were similar in many respects to those found in the wild. For example, during the dry season, fish in the upper reaches of the river system become confined to small and isolated pools that can range in surface area from 1 to 10 m^2^ and contain a varying number of fish that will be together for a period of several months (Griffiths & Magurran 1997). Importantly, across natural populations of guppies, there is great variation in the degree to which females experience sexual harassment, which can be linked to, for example, variation in the relative proportion of males (Head & Brooks 2006) and other ecological pressures (Magurran & Seghers 1994b). This provides an exciting opportunity for future work to test the hypothesis that high levels of sexual harassment will drive increased swimming performance in the wild.

A remaining question is why all females do not improve the efficiency of movement when they clearly have the capacity to do so. A possible explanation is that the ‘training’ required for increased exercise economy requires a substantial investment of time and energy: HM females are being forced into performing extra bouts of swimming, which in the wild would likely interfere with the ability to forage and could attract predators (Magurran & Seghers 1994a; Rowe 1994). It is notable that the difference in locomotory efficiency between LM and HM females appears to be most obvious at intermediate to high speeds. Therefore, fish that spend the majority of time swimming relatively slowly not only will not have the opportunity to display plasticity in swimming physiology or mechanics, but they may not need to. There could also be a functional trade‐off between increased aerobic efficiency and anaerobic capacity (Chappell & Odell 2004; Marras *et al*. 2010), as unexpectedly, HM females tended to have a reduced anaerobic capacity (as measured by EPOC) as compared to LM females. Differences in the proportions of red and white muscle fibres could be the basis of such a trade‐off, an area which would require further study.

In summary, female guppies exposed to high levels of male harassment showed increased swimming efficiency, spending less energy to move a given speed and distance. A main mechanism for this difference appears to be swimming mechanics – females that had previously been exposed to high levels of male harassment moved using less pectoral fin‐assisted swimming. These results show that sexual harassment can result in plastic changes in the routine energy expenditure and physiology of females. The implications for such changes for population dynamics, sexual selection and interactions with foraging or predator avoidance will be important avenues for future study.

## Supporting information


**Lay Summary**

**Fig. S1.** Comparison the remaining aerobic scope, after accounting for the costs of locomotion, while swimming at various speeds for females guppies reared for 5 months with exposure to varying levels of male harassment (*n* = 10 per treatment). Filled circles = low harassment females; open circles = high harassment females.

**Fig. S2.** Relationship between critical swimming speed (*U*_crit_) and mean pectoral fin use during aerobically powered swimming in female guppies reared for 5 months with exposure to varying levels of male harassment. Each data point represents one individual (*n* = 8 per treatment). Filled circles = low harassment females; open circles = high harassment females.

**Table S1.** Morphological comparisons between female guppies reared for several months with exposure to varying levels of male harassment (*n* = 8 per treatment).
