# Supplementary material for: Male sexually coercive behaviour drives increased swimming efficiency in female guppies
Source: Funct Ecol. 2015 Aug 24;30(4):576–83. doi: 10.1111/1365-2435.12527 (PMC4949636; doi:10.1111/1365-2435.12527)
Supplement: Supplementary file 1 — Lay Summary [file FEC-30-576-s001.pdf]

Functional Ecology

British Ecological Society

Female guppies reduce energetic costs of being harassed by males by becoming more efficient swimmers

Shaun S. Killen, Darren P. Croft, Karine Salin and Safi K. Darden

Females and males of the same species are often in conflict over the frequency and timing of matings. Typically male reproductive success is limited by access to females and males of many species will try to overcome this using a number of behaviours, such as chasing and even attacking females in an attempt to gain a mating. These types of behaviours are considered sexually harassing as males are attempting to coerce females into mating with them. Females can spend a lot of energy avoiding males in these situations and can even be injured. To reduce these costs, one possibility is that females may be able to change their own behaviour or physiology in ways that reduce the negative energetic consequences of harassment, or allows them to more easily escape male coercion.

We studied this likelihood in a laboratory setting by housing female Trinidadian guppies *(Poecilia reticulata)* for several months with varying levels of male harassment that they would normally encounter in the wild. In the wild male guppies spend a large portion of their time chasing and harassing females in an attempt to mate with them. Females can attempt to avoid this harassment by rapidly swimming away from the male during pursuits.

After five months, females exposed to higher levels of harassment were able to swim much more efficiently, using less energy to swim at a given speed compared to those exposed to lower levels of harassment. It seems that prolonged increases in high-intensity swimming in females, caused by male harassment, leads to changes in the physiology or swimming mechanics of individual fish, which reduce costs of swimming. Indeed, females that experienced lower levels of harassment spent more time swimming with their pectoral fins extended, an indicator of an inefficient swimming technique.

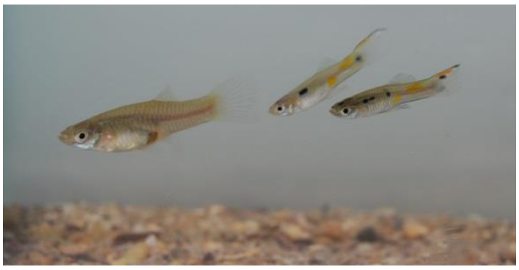

Image provided by authors.

These results show that female guppies can reduce the energetic costs of male sexual harassment through changes in their swimming physiology or technique. Increased swimming efficiency or performance could also allow female guppies to escape male coercion more easily, giving them more control over matings. An exciting opportunity now exists to examine the extent to which this phenomenon occurs in the wild.
