# Supplementary figures and images for: Male sexually coercive behaviour drives increased swimming efficiency in female guppies
Source: Funct Ecol. 2015 Aug 24;30(4):576–83. doi: 10.1111/1365-2435.12527 (PMC4949636; doi:10.1111/1365-2435.12527)

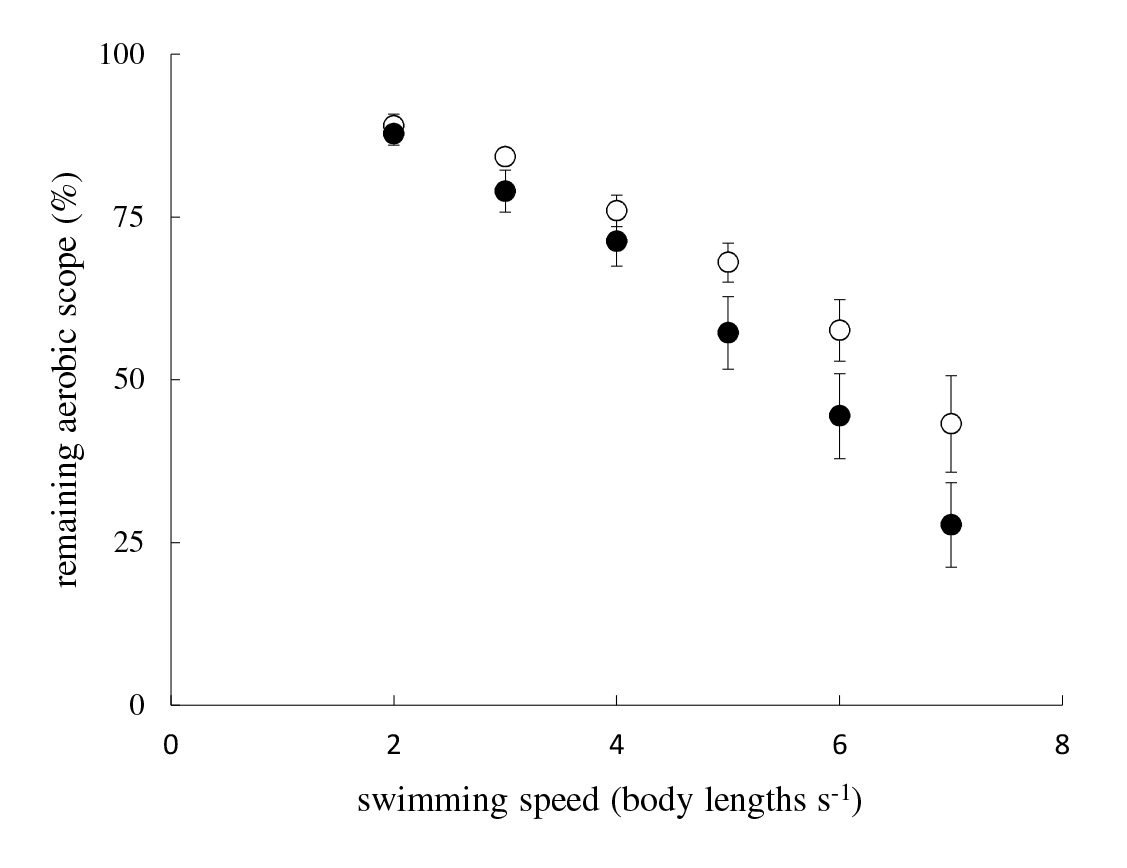

Supplement: Supplementary file 2 — Fig. S1. Comparison the remaining aerobic scope, after accounting for the costs of locomotion, while swimming at various speeds for females guppies reared for 5 months with exposure to varying levels of male harassment (n = 10 per treatment). Filled circles = low harassment females; open circles = high harassment females. [file FEC-30-576-s002.tif]

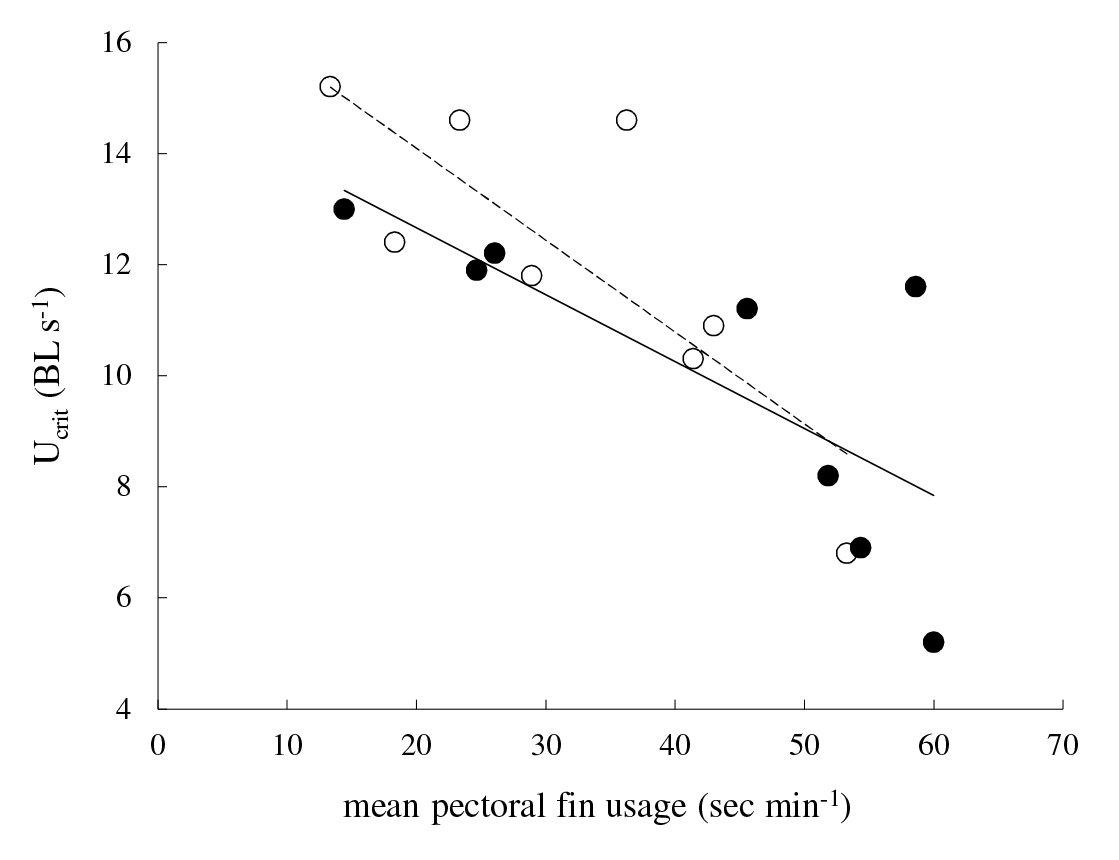

Supplement: Supplementary file 3 — Fig. S2. Relationship between critical swimming speed (U crit) and mean pectoral fin use during aerobically powered swimming in female guppies reared for 5 months with exposure to varying levels of male harassment. Each data point represents one individual (n = 8 per treatment). Filled circles = low harassment females; open circles = high harassment females. [file FEC-30-576-s003.tif]
