# Supplementary material for: Male sexually coercive behaviour drives increased swimming efficiency in female guppies
Source: Funct Ecol. 2015 Aug 24;30(4):576–83. doi: 10.1111/1365-2435.12527 (PMC4949636; doi:10.1111/1365-2435.12527)
Supplement: Supplementary file 4 — Table S1. Morphological comparisons between female guppies reared for several months with exposure to varying levels of male harassment (n = 8 per treatment). [file FEC-30-576-s004.docx]

**Table s1.** Morphological comparisons between female guppies reared for several months with exposure to varying levels of male harassment (n = 8 per treatment).

**Variable low harassment high harassment F p**

wet mass (g) 0.502±0.0812 0.492±0.106 0.085 0.772

standard length (mm) 29.384±1.132 29.405±1.791 0.002 0.967

body depth (mm) 7.525±0.686 7.380±0.775 0.780 0.383

peduncle depth (mm) 4.911±0.266 4.908±0.323 0.005 0.945

caudal length (mm) 8.435±0.664 8.239±0.604 1.170 0.287

caudal height (mm) 6.394±0.710 6.452±0.755 0.287 0.595

caudal surface area (mm^2^) 50.292±6.999 50.531±6.997 0.009 0.924

caudal aspect ratio 0.801±0.110 0.827±0.110 0.557 0.460

eye diameter (mm) 2.135±0.135 2.115±0.162 0.272 0.605
